# Alcohol consumption patterns and the risk of sarcopenia: a population-based cross-sectional study among chinese women and men from Henan province

**DOI:** 10.1186/s12889-022-14275-6

**Published:** 2022-10-11

**Authors:** Junya Zhai, Baihui Ma, Jin Qin, Quanjun Lyu, Pipasha Khatun, Rui Liang, Minghua Cong, Lijun Guo, Yongxia Kong

**Affiliations:** 1 Department of Clinical Nutrition, Affiliated Cancer Hospital of Zhengzhou University, Henan Cancer Hospital, Zhengzhou, China; 2 Department of Nutrition, The First Affiliated Hospital of Zhengzhou University, Zhengzhou, China; 3 Department of Nutrition and Food Hygiene, College of Public Health, Zhengzhou University, Zhengzhou, Henan China; 4 Department of Comprehensive Oncology, National Clinical Research Center for Cancer/Cancer Hospital, National Cancer Center, Chinese Academy of Medical Sciences and Peking Union Medical College, Beijing, China

**Keywords:** Alcohol consumption patterns, Drinking frequency, Sarcopenia, Association

## Abstract

**Objectives:**

Sarcopenia is a clinically relevant syndrome with health, social, and economic implications. Alcohol consumption is one of the risk factors for sarcopenia, but it has not been fully investigated in Chinese populations. The purpose of the present study was to assess the relationship between alcohol consumption patterns (including the volume and frequency of alcohol consumption) and sarcopenia or its elements among Chinese women and men from Henan Province.

**Method:**

A cross-sectional study was designed to collect information about nutrition and health in Henan Province, China, and a total of 680 individuals were studied. Sarcopenia was defined according to the Asian Working Group for Sarcopenia (AWGS) criteria updated in 2019. Alcohol consumption was calculated in grams per day and divided into three groups. Drinking frequency was divided into four groups. We assessed the likelihood that sarcopenia was associated with drinking patterns using multiple logistic regression analysis by odds ratios (ORs) with 95% confidence interval (CIs) after multiple adjustments.

**Results:**

We found that men who consumed > 25 g of ethanol per day were approximately three times more likely to have low muscle mass (OR, 3.99; 95% CI, 1.10–17.05) or low grip strength (OR, 3.39; 95% CI, 1.33–9.92) than nondrinkers after multiple adjustments. In addition, males who consumed alcohol more than 4 times per week were approximately threefold to fivefold more likely to have low muscle mass (OR, 4.99; 95% CI, 1.24–25.55) or low grip strength (OR, 3.37; 95% CI, 1.40–8.74) than nondrinkers. Unfortunately, we did not find a relationship between alcohol consumption patterns and sarcopenia or gait speed among males, and there was no association between alcohol consumption patterns and sarcopenia or any elements.

**Conclusion:**

Heavy alcohol consumption and frequent drinking are important risk factors for low muscle mass and muscle strength in Chinese men from Henan province.

## Introduction

The overall life expectancy of humans is dramatically increasing worldwide, leading to a surge in the elderly population. Aging is characterized by skeletal muscle mass decline by 1–2% annually after the age of 50, in concert with a decline in strength of 1.5% per year that accelerates to 3% annually after the age of 60 [1]. The characteristics of the decrease in muscle mass, muscle strength, and muscle efficiency with aging are often referred to as “sarcopenia” [2]. The prevalence of sarcopenia varies widely, ranging from 5.5 to 25.7% based on the Asian Working Group for Sarcopenia (AWGS) 2014 criteria for Asian populations [3]. Sarcopenia is considered to be an independent risk factor for various adverse outcomes, including osteoporosis, falls, reduced cardiopulmonary function, metabolic syndrome, and insulin resistance, and eventually leads to disability, longer lengths of stay at the hospital, readmission and death [4]. Thus, exploring the risk factors could be an effective solution for identifying early markers for sarcopenia prevention.

Alcohol consumption is responsible for 5.1% of the global burden of disease and injury [5]. Drinking alcohol is a widely accepted traditional Chinese cultural behavior. The drinking rate was 53.8% and 12.2% for men and women, respectively, based on the 2010–2012 national China Nutrition and Health Survey [6], which is increasing faster. [7]. Accordingly, drinking alcohol has become a serious problem that should be addressed.

Alcohol consumption is one of the modifiable behavioral factors that accelerates sarcopenia [8], which has attracted the attention of researchers. Some experimental studies among animals have demonstrated that alcohol consumption inhibits the synthesis of skeletal muscle proteins [9–11]. In humans, this relationship remains controversial, although a positive relationship has been reported between alcohol consumption and sarcopenia in the general population [12, 13]. In addition, the results of a recently reported meta-analysis did not support alcohol consumption as a risk factor for sarcopenia [14]. However, studies included in that meta-analysis were not designed considering the relationship between alcohol consumption and sarcopenia as the primary end point [15, 16].

In China, the relationship between alcohol consumption patterns and the risk of sarcopenia has not been fully investigated. Some studies focused on drinking frequency [17, 18], whereas others evaluated whether people should drink or not [19, 20], and most of them did not adjust for confounding factors in the analysis. In addition, no previous research has evaluated the effect of different alcohol consumption patterns as the primary end point on sarcopenia in Chinese populations. This is important because, except for drinking frequency, total alcohol intake may influence health outcomes. Accordingly, we assessed the association between patterns of alcohol consumption and the risk of sarcopenia using information from a cross-sectional study of general Chinese adults from Henan province.

## Participants and methods

### Study design and population

Our study population included residents aged 25–75 years from three communities in Henan, China. The diet, lifestyle, and anthropometry information of the participants was collected for investigation from January to November 2020, after excluding incomplete data and implausible energy intake. We excluded abstainers (n = 12) from the analysis because the number of people in this group was relatively small and because of the presumed presence of “sick quitters” (individuals who stopped drinking due to illness) among them [21]. We also excluded people with missing information (n = 75) or with conflicting answers regarding the amount and frequency of alcohol intake (n = 18). In all, 680 people (411 males and 269 females) were eligible for this study.

This study was approved by the Ethical Review Committee, and written informed consent was obtained from all participants (Protocol 2020–KY-066).

### Sarcopenia assessment

Sarcopenia was measured by the recommended revised diagnostic algorithm of the AWGS [3]. Lean muscle mass in this study was measured by bioelectrical impedance analysis (Inbody 570, BioSpace, Seoul, Korea). All participants changed into paper gowns and were asked to remove all jewelry and other personal effects that could interfere with the examination. The appendicular skeletal muscle mass index (SMI) was calculated by the following formula: (lean mass from arm and leg)[kg]/height [m]^2^). Low muscle mass was defined by an SMI below 5.7 kg/m2 in females and below 7.0 kg/m^2^ in males. Muscle strength was assessed by grip strength, measured using a dynamometer (EH101; Camry, Zhongshan, China). The participants stood with their arms and wrists by the sides of the body and were asked to exert maximum grip with each hand. Tests were performed on two independent occasions, and the better of 2 results were chosen for analysis. The criteria were 28 kg and 18 kg for males and females, respectively. Usual gait speed (GS) on a 6 m course was used as an objective measure of physical performance, which was defined as < 1.0 m/s. Sarcopenia was identified by low muscle mass plus muscle strength or physical performance, and severe sarcopenia was diagnosed when all three criteria of the definition were met [3]. Because of the small sample size of this study, the sarcopenia stage (including severe sarcopenia) and its elements (low muscle mass, muscle strength, and physical performance) were analyzed.

### Alcohol consumption assessment

Usual consumption of alcoholic beverages in the previous year was estimated with a validated diet history, which collected information on the beverage type (beer, wine, and hard liquor), the amount and frequency of intake, and used sets of food containers to help quantify the amount. Alcohol consumption was calculated in grams per day by multiplying the average frequency (times per day) by the usual consumption amount of each beverage and its average pure ethanol content (5.0 g for 100 g of beer, 12.0 g for 100 g of wine [22], and 40.0 g for 100 g of hard liquor) [23]. According to the dietary guidelines for Chinese residents [24], the recommendation for alcohol consumption is 25 g of pure ethanol per day for men and 15 g of pure ethanol per day for women. Accordingly, the participants were divided into three groups depending on the amount of alcohol consumed per day (nondrinker, light-to-moderate drinker (1–25 g/day), and heavy drinker (> 25 g/day)) for males and three groups (nondrinker, light-to-moderate drinker (1–15 g/day), and heavy drinker (> 15 g/day)) for females. The frequency of drinking was divided into four groups: nondrinker, < once/week, 1–4 times a week, and > 4 times a week.

### Assessment of other variables

Data were collected by a general questionnaire and through two nonconsecutive 24-h dietary recalls. To help the respondents answer accurately, the 24-h dietary recalls were conducted face to face with the aid of food models. The average daily food intake and protein intake were analyzed by a nutrition calculator (Nutrition and Diet Management System of Traditional Chinese Medicine Combining with WesternMedcine software, NCCW software), which was calculated based on the China food composition Table [21]. Weight and height were measured by experienced investigators. The general questionnaire assessed age, sex, educational attainment, occupational status, marital status, income level, smoking status, snap frequency, and so on. Physical activity was collected through the Chinese version of the international physical activity questionnaire (IPAQ) [25], which appeared to have acceptable reliability and validity. Moderate-vigorous physical activity (Met-h/day, MET, metabolic equivalent of task) was calculated for each individual according to guidelines for Chinese residents [24].

### Statistical analysis

Data analysis was performed by using SAS statistical software, version 9.3 (SAS Institute, Cary, NC, USA), for all data analyses. A p value < 0.05 was considered statistically significant.

The normality of continuous variables was first tested by the Shapiro‒Wilk test and Kolmogorov‒Smirnov test. According to the data distribution, continuous variables were described by parametric tests or nonparametric tests. Categorical variables are provided as percentages (%), and chi-squared tests were used to determine whether any significant differences existed between groups.

We assessed the likelihood that sarcopenia was associated with drinking patterns (alcohol consumption, drinking frequency) using multiple logistic regression analysis by odds ratios (ORs) with 95% confidence interval (CIs). In the analysis, Model 1 was the crude model, and Model 2 adjusted for potential confounders (age, BMI, marital status, food intake, smoking, nap frequency, and moderate-vigorous physical activity).

## Results

Data from 680 participants were used for analysis using the AWGS algorithm, which was updated in 2019. Table 1 shows the characteristics of the study participants according to their daily alcohol consumption by sex. Among males, 411 individuals with a mean age of 60 years were included in our study. Of these, 218 (53.0%) and 94 (22.9%) participants were defined as light-to-moderate drinkers and heavy drinkers, respectively. The proportions of Chinese males who were current smokers and married were higher at higher levels of alcohol intake. The prevalence of low grip strength among heavy drinkers was approximately threefold higher than that among nondrinkers. Among females, 269 participants with a mean age of 58 years were included in the analysis. Of these, 117 (53.2%) and 9 (3.3%) participants were defined as light-to-moderate drinkers and heavy drinkers, respectively. The variables of age, educational level, and grip strength were quite different according to alcohol consumption levels (Table 1).

**Table 1:** Baseline characteristics according to alcohol consumption among males (n = 411)^1^ and females (n = 269)^2^

	Alcohol consumption (among males)^1^				Alcohol consumption (among females)^2^			
	Never 99(24.1%)	Light-to-moderate 218(53.0%)	Heavy 94(22.9%)	P	Never 143(53.2%)	Light-to-moderate 117(43.5%)	Heavy 9(3.3%)	P
Ethanol	0	6.66 ± 6.70	61.02 ± 52.15	<0.0001	0	6.39 ± 6.49	60.12 ± 51.11	<0.0001
Age (y)	60.11 ± 9.51	61.07 ± 7.73	60.12 ± 7.47	0.492	57.92 ± 9.42	53.12 ± 8.79	55.44 ± 11.05	0.002
BMI, kg/m2	25.71 ± 3.41	25.80 ± 6.46	25.73 ± 3.51	0.986	24.99 ± 3.25	24.26 ± 2.77	26.67 ± 3.76	0.032
Physical activity (MET-h/day)	8.25 ± 6.01	7.59 ± 7.39	7.08 ± 4.94	0.470	8.05 ± 8.70	8.68 ± 6.84	8.05 ± 7.32	0.662
Nap frequency	4.01 ± 2.80	4.21 ± 2.59	4.19 ± 2.49	0.824	3.65 ± 2.58	3.82 ± 2.59	2.94 ± 2.24	0. 607
Food intake (kcal)	1608 ± 527	1622 ± 441	1606 ± 473	0.954	1372 ± 433	1399 ± 388	1463 ± 352	0.758
Married				0.045				0.593
Yes	92(92.9%)	209(95.9%)	83(88.3%)		127(88.8%)	104(88.9%)	7(77.8%)	
No	7(7.1%)	9(4.1%)	11(11.7%)		16(11.2%)	13(11.1%)	2(22.2%)	
Education level				0.871				0.019
≤Primary school	7(7.1%)	20(9.2%)	8(8.5%)		33(23.1%)	17(14.5%)	1(11.1%)	
Secondary school	72(72.7%)	145(66.5%)	64(68.1%)		91(63.6%)	66(56.4%)	5(55.6%)	
University	20(20.2%)	53(24.3%)	22(23.4%)		19(13.3%)	34(29.1%)	3(33.3%)	
Income				0.115				0.068
Low	25(25.3%)	32(14.7%)	20(21.3%)		28(19.6%)	19(16.2%)	1(11.1%)	
Middle	50(50.5%)	132(60.6%)	46(48.9%)		88(61.5%)	62(53.0%)	7(77.8%)	
High	24(24.2%)	54(24.8%)	28(29.8%)		27(18.9%)	36(30.8%)	1(11.1%)	
Smoking				0.004				0.290
Yes	44(44.4%)	109(50.0%)	66(70.2%)		5(3.5%)	3(2.6%)	1 (11.1%)	
No	34(34.4%)	66(30.3%)	20(21.3%)		135(94.4%)	114(42.7%)	8(88.9%)	
Abstainers	21(21.2%)	43(19.7%)	8(8.5%)		3(2.1%)	0(0%)	0(0%)	
Sarcopenia				0.472				0.708
Yes	5(5.1%)	19(8.7%)	6(6.4%)		11(7.7%)	9(7.7%)	0(0%)	
No	94(94.9%)	199(91.3%)	88(93.7%)		132(92.3%)	108(92.3%)	9(100%)	
SMI^4^				0.627				0.596
Yes	10(10.1%)	20(9.2%)	6(6.4%)		14(9.8%)	13(11.1%)	0(0%)	
No	89(89.9%)	198(90.8%)	88(93.6%)		129(90.2%)	104(88.9%)	9(100%)	
Grip strength				0.002				0.049
Yes	20(20.2%)	20(9.2%)	6(6.4%)		28(19.6%)	12(10.3%)	0(0%)	
No	79(79.8%)	198(90.1%)	88(93.6%)		115(80.4%)	105(89.7%)	9(100%)	
Gait speed				0.099				0.254
Yes	71(71.7%)	185(84.9%)	73(77.7%)		100(69.9%)	92(78.6%)	6(66.7%)	
No	28(28.3%)	33(15.1%)	21(22.3%)		43(30.1%)	25(21.4%)	3(33.3%)	

Note: ^1^ Participants were classified into the following categories of alcohol consumption: never, light-to-moderate (≤ 25 g of ethanol) and heavy (> 25 g of ethanol). ^2^ Participants were classified into the following categories of alcohol consumption: never, light-to-moderate (≤ 15 g of ethanol) and heavy (> 15 g of ethanol). ^3^ For continuous variables, the means ± SDs are provided. One-way ANOVAs were selected to determine whether there were any significant differences. Categorical variables are provided as numbers and percentages (%), and chi-squared tests were used to determine whether any significant differences existed between groups. ^4^ SMI (skeletal muscle mass index)

Table 2 presents the association between alcohol consumption patterns (three levels of volume of drinking and frequency) and the risk of sarcopenia and its elements among Chinese males and females. Among males, we found a positive association between alcohol consumption patterns and the risk of low muscle mass and low muscle strength. Those who consumed > 25 g of ethanol per day were approximately three times more likely to have low muscle mass (OR, 3.99; 95% CI, 1.10–17.05) or low grip strength (OR, 3.39; 95% CI, 1.33–9.92) than nondrinkers after adjustment for age, marital status, smoking, BMI, food intake, physical activity and nap frequency. Those who consumed alcohol more than 4 times per week were approximately threefold to fivefold more likely to have low muscle mass (OR, 4.99; 95% CI, 1.24–25.55) or low grip strength (OR, 3.37; 95% CI, 1.40–8.74) than nondrinkers after multiple adjustments. Unfortunately, we did not find a relationship between alcohol consumption patterns and sarcopenia or gait speed among males. Among females, because of the small number of drinkers, we combined the light-to-moderate drinker group and heavy drinker group. That is, there were two groups (never drinker and current drinker) for the analysis of alcohol consumption and drinking frequency. However, there was no separate relationship of alcohol consumption and sarcopenia or its elements with alcohol consumption and drinking frequency.

**Table 2:** Association between alcohol consumption patterns and the risk of sarcopenia and its elements among males and females

	Sarcopenia		SMI^1^		Grip strength		Gait speed	
	Model 1^4^	Model 2^5^	Model 1^4^	Model 2^5^	Model 1^4^	Model 2^5^	Model 1^4^	Model 2^5^
	Odds ratio	Odds ratio	Odds ratio	Odds ratio	Odds ratio	Odds ratio	Odds ratio	Odds ratio
Males								
Alcohol consumption^2^								
Never	Reference							
Light-to-moderate	0.56(0.18, 1.43)	0.38 (0.10, 1.18)	1.11(0.48,2.42)	1.00(0.38,2.46)	2.88(1.49,5.56)	2.94(1.42,6.20)	0.63(0.35,1.14)	0.62(0.33,1.20)
Heavy	0.78(0.22, 2.68)	1.20(0.24, 5.90)	1.65(0.59,5.02)	3.99(1.10,17.05)	3.81(1.61,10.09)	3.39(1.33,9.92)	1.02(0.53,1.97)	0.97(0.48,1.99)
Drinking frequency								
Never	Reference							
< Once per week	0.30(0.07,0.97)	0.32(0.07,1.12)	0.98(0.38,2.42)	0.73(0.24,2.08)	2.36(1.14,5.01)	2.45(1.06,5.90)	0.41(0.20,0.83)	0.47(0.21,1.02)
1–4 times per week	0.35(0.08,1.32)	0.28(0.06,1.12)	1.04(0.39,2.78)	0.90(0.28,2.85)	5.80(2.25,18.00)	4.21(1.56,13.45)	0.56(0.27,1.12)	0.59(0.27,1.28)
> 4 times per week	1.79(0.34,10.28)	1.38(0.23,8.31)	2.00(0.66,6.73)	4.99(1.24,25.55)	3.09(1.38,7.46)	3.37(1.40,8.74)	1.08(0.57,2.06)	1.09(0.55,2.19)
Females^3^								
Never drinker	Reference							
Current drinker	1.11(0.44,2.84)	0.78(0.24,2.55)	0.96(0.43,2.16)	0.78(0.28,2.21)	2.31(1.15,4.93)	1.51(0.67,3.54)	0.66(0.38,1.15)	0.71(0.38,1.33)

Note: ^1^ SMI (skeletal muscle mass index). ^2^ Never drinker (nondrinker), light-to-moderate drinker (1–25 g/day), and heavy drinker (> 25 g/day)) for males. ^3^ Because of the small number of drinkers, we combined the light-to-moderate drinker group and the heavy drinker group. That is, there were two groups (never drinker and current drinker) for the analysis of alcohol consumption and drinking frequency. ^4^ Model 1: Crude model. ^5^ Model 2: Adjusted for age, marital status, BMI, nap frequency (continuous), food intake (continuous), moderate-vigorous physical activity (MET-h/d, continuous), and smoking (Yes/no)

## Discussions

In this cross-sectional study, we examined the association between alcohol consumption patterns and the prevalence of the risk of sarcopenia among Chinese males and females from Henan Province. We concluded that higher alcohol intake (including volume and frequency) was linked to a tendency toward a higher risk of low muscle mass and low muscle strength after multiple adjustments, but no relation was found between alcohol consumption patterns and sarcopenia or gait speed among males. In addition, the relationship between alcohol consumption patterns and sarcopenia or its elements was not observed among females.

Alcohol consumption is one of the leading causes of disease burden [26] In human studies, although the relationship between alcohol consumption and sarcopenia has been reported in the general population, related studies are still sparse, and their results are still controversial [12–14]. Among males, our findings showed that there was no relation between alcohol drinking patterns and sarcopenia or gait speed. However, we found that alcohol drinking patterns were positively associated with low muscle mass and low grip strength after multiple adjustments among males. Our findings were in line with previous studies that showed that high alcohol consumption was associated with a greater decline in the skeletal muscle index among older Brazilian people [27]. Moreover, a population-based prospective study showed a significant positive association between alcohol consumption and a decline in muscle strength, and this association did not change over the 2-year period among Japanese individuals [28]. The phenomenon of alcohol drinking patterns and a decline in muscle strength and the skeletal muscle index is known as “chronic alcoholic myopathy” [29], which manifests as a decrease in muscle mass and a reduced cross-sectional area for type II fiber-rich muscle [30, 31]. There have been many reports on the underlying molecular mechanism responsible for alcoholic myopathy. Studies have indicated that long-term alcohol consumption results in a protracted imbalance in protein homeostasis by impairing translation initiation in muscle by altering the activities of several eukaryotic initiation factors [32] or reducing protein/DNA ratios, a fact associated with lower myofibrillary Ca^2^-ATPase activity [33]. Moreover, alcohol intake induces defects in the insulin signal transduction pathway as a decreased ability of maximal stimulating doses of insulin growth factor (IGF)-1 to upregulate muscle protein synthesis [34]. Skeletal myopathy is worthy of attention because it may occur in as many as one-third of high alcohol consumers and may lead to a significant loss of muscle strength and function [35]. Poor muscle strength has also been associated with a high prevalence of chronic diseases [36] and a higher risk of mortality in older populations [37]. Although alcohol consumption is not known as a direct cause of sarcopenia, studies demonstrating the adverse effects of alcohol on muscle mass suggest that chronic alcohol consumption may promote the loss of muscle mass and strength. Therefore, reducing alcohol consumption may serve as a strategy for the prevention of the decline in muscle mass and muscle strength.

We found that the association between alcohol drinking patterns and sarcopenia or its elements was different for males and females. Several explanations may account for a possible interaction between sex and alcohol consumption patterns in this study. The number of cases was substantially lower among females than among males. In addition, the volume and frequency of alcohol consumption among Chinese women was quite low, with a mean of 1.1 g of ethanol daily for quantity and a mean of 0.44 times per week for frequency, whereas it was 17.3 g and 2.41 times per week among men, respectively. We cannot exclude the possibility that this result is related to low alcohol consumption. Finally, differences in alcohol pharmacokinetics [38] and beverage preferences between sexes may be another explanation. One article stated that a preference for a specific type of alcoholic beverage (wine or other) was considered when such a drink accounted for more than 80% of alcohol intake [39]. The preferred beverage was wine among females, which may be more beneficial than drinking beer or spirits [40], whereas liquor was favored by the males in this study. However, the results for women are less certain and warrant further study.

This study had several strengths and limitations. First, this was the first study to explore the association between alcohol consumption and sarcopenia in the general Chinese population. Additionally, sarcopenia was defined according to the updated standard criteria in 2019. An important strength was the definition of alcohol, which was divided into groups according to the dietary guidelines for Chinese residents, which provides a sound basis for drinking guidelines for the Chinese population. 

Among the main limitations was that alcohol consumption was self-reported, so there may be recall error and social desirability bias. In addition, this study regarding alcohol intake and sarcopenia failed to investigate the dose‒response relationship. Moreover,  considering the small sample size, we have not analysed by different age group. It is an urgent need to continue to collect samples and to further explore the relationship between alcohol consumption and sarcopenia at different age and gender level. Finally, our study was a cross-sectional study, and a causal relationship between alcohol consumption and sarcopenia or its elements should be investigated in a follow-up study.

## Conclusion

These data showed heavy alcohol consumption and frequent drinking was associated with an increased risk of low muscle mass and muscle strength in Chinese men from Henan province. . Efforts to reduce alcohol consumption and drinking frequency among males in China should be pursued as part of comprehensive lifestyle modification approaches for the prevention and treatment of low muscle mass and muscle strength.

## Data Availability

The datasets used and/or analyzed during the current study are available from the corresponding author upon reasonable request.
